# Overlapping *ATP2C1* and *ASTE1* Genes in Human Genome: Implications for SPCA1 Expression?

**DOI:** 10.3390/ijms14010674

**Published:** 2013-01-04

**Authors:** Massimo Micaroni, Lorenzo Malquori

**Affiliations:** 1 School of Biosciences, University of Exeter, Exeter, England EX4 4QD, UK; 2 Division of Molecular Cell Biology, Institute for Molecular Bioscience, the University of Queensland, 306 Carmody Road, St. Lucia, Brisbane QLD 4072, Australia; 3 Faculty of Health Sciences, The Southbank Institute of Technology, 66 Ernest Street, South Brisbane QLD 4101, Australia; E-Mail: l.malquori@gmail.com

**Keywords:** *ASTE1*, *ATP2C1*, ATPase Ca^2+^ pump, gene expression, Golgi apparatus, Hailey-Hailey disease, intracellular membrane trafficking, SPCA1

## Abstract

The *ATP2C1* gene encodes for the secretory pathway calcium (Ca^2+^)-ATPase pump (SPCA1), which localizes along the secretory pathway, mainly in the *trans*-Golgi. The loss of one *ATP2C1* allele causes Hailey-Hailey disease in humans but not mice. Examining differences in genomic organization between mouse and human we speculate that the overlap between *ATP2C1* and *ASTE1* genes only in humans could explain this different response to *ATP2C1* dysregulation. We propose that *ASTE1*, overlapping with *ATP2C1* in humans, affects alternative splicing, and potentially protein expression of the latter. If dysregulated, the composition of the SPCA1 isoform pool could diverge from the physiological status, affecting cytosolic Ca^2+^-signaling, and in turn perturbing cell division, leading to cell death or to neoplastic transformation.

## 1. Introduction

**Figure 1:**
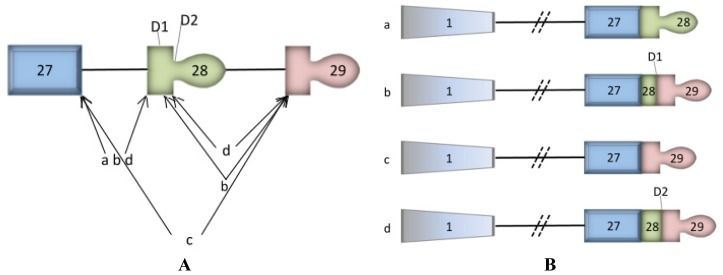

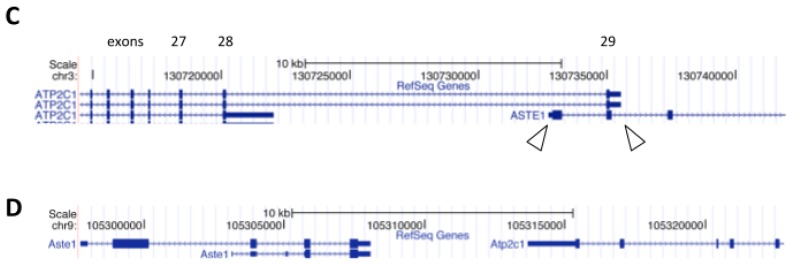
The *ATP2C1* gene transcription site overlaps with the *ASTE1* site in the human but not mouse genome. (**A**) This panel represents the 3′ organization of the *ATP2C*1 gene providing an overview of alternative splicing events. Exons are represented by boxes, with wide boxes depicting the open reading frame. The thin horizontal line represents the position of introns. The internal 5′ donor splice sites, D1 and D2, are also represented. The arrows illustrate the splicing patterns generating splice variants *ATP2C1a*–*d*. The exons positions according to the NCBI reference sequence NG_007379.1 are: exon 27 (109929–110070), exon 28 (*ATP2C1a*: 111631–113613; *ATP2C1b*: 111631–111725; *ATP2C1d*: 111631–111725), exon 29 (126563–127123). (**B**) The *ATP2C1a*–*d* splice variants are schematically represented. (**A**) and (**B**) are modified from Fairclough *et al.* [1]. (**C**,**D**) Genomic analysis using the UCSC Genome Browser (available online: http://genome.ucsc.edu (accessed on 5 December 2012)) revealed a partial overlapping (empty arrowheads) of *ATP2C1* with *ASTE1* present in the human genome (chromosome 3) (**C**), but not in the murine genome (chromosome 9) (**D**). The *ASTE1* open reading frame is oriented in the opposite direction with respect to that of *ATP2C1* in both genomes.

The human *ATP2C1* gene transcript, encoding for the secretory pathway calcium (Ca^2+^)-ATPase pump type 1 (SPCA1), is alternatively spliced. Although there has been some confusion about the various splice variants, Fairclough *et al.* presented a unifying study describing four isoforms [1]. The corresponding proteins are termed SPCA1a-d and only differ in their carboxyl termini (Figure 1A,B). Three splice variants SPCA1a,b, and d are functional whereas SPCA1c, which is truncated within the last transmembrane segment, is nonfunctional and rapidly degraded [2]. Furthermore, the terminal exon of the human *ATP2C1* gene overlaps with the *ASTE1* gene on the opposing strand and whose open reading frame is transcribed towards *ATP2C1* (Figure 1C) [3]. This transcriptional overlap is not present in the mouse genome (Figure 1D), according to the present literature and experimental data [4], where no alternative splicing has been detected so far.

## 2. Mutations in *ATP2C1* Cause Hailey-Hailey Disease in Humans but not Mice

Hailey-Hailey disease (HHD; also known as familial benign pemphigus) is a human autosomal-dominant skin disease caused by the loss of one functional copy of the *ATP2C1* gene [5,6], characterized by acantholysis (a disruption of cell-cell contacts) in the suprabasal layers of the skin [6]. SPCA1 protein levels in HHD keratinocytes are reduced to about half of normal levels, and Golgi Ca^2+^ handling is impaired [7]. Normal function of SPCA1 contributes to correct trafficking of cellular adhesion proteins, and defects in normal expression levels of SPCA1 lead to the HHD symptoms by affecting intracellular membrane trafficking [8–10]. Surprisingly, it has been recently reported that human SPCA1-deficient keratinocytes displayed high levels of tight junction proteins claudins 1 and 4, while desmosomal components were not affected [11], indicating that tight junction and desmosomal proteins are regulated differently. In humans, a low incidence of squamous cell tumors has been reported in HHD [12,13] but it is unclear whether this is a chance association or it is caused by the reduction in Ca^2+^ pump levels and/or activity.

A gene-targeted mouse model for SPCA1 has been used to analyze the phenotype resulting from heterozygous and homozygous null mutations; SPCA1 null embryos undergo a substantial degree of structural development and survive until gestation day 10.5 [14]. However, embryonic tissues exhibited a high incidence of apoptosis and ultra-structural evidence of severe Golgi stress, thus establishing SPCA1 deficiency as an example of a condition that causes Golgi stress [14]. Heterozygous mutants exhibited no evidence of HHD but did develop squamous cell tumors [14]. The effects of SPCA1 deficiency is consistent with a model in which species differences in the balance between pro-survival and pro-apoptotic responses of keratinocytes to secretory pathway stress favor development of cancer in mice and acantholytic skin disease in humans.

Interestingly, while comparing the human and murine genomes, we observed that the 3′UTR of *ATP2C1* overlaps with the 3′UTR antisense of *ASTE1* (which encodes Asteroid1, of unknown function) in human, but not in mouse (Figure 1C,D). We speculate that this difference could explain the different pathologies caused by the loss of one allele of *ATP2C1* (with consequent lower levels of SPCA1 protein) in human *vs.* mouse. The *ATP2C1/ASTE1* partial overlap (Figure 1C, arrowheads) could represent a human-specific regulatory mechanism; when *ASTE1* is mutated (e.g., in human colorectal cancers [15]) the SPCA1 protein could accumulate, potentially leading to secretory pathway abnormalities and eventually neoplastic transformation. Conversely, when *ATP2C1* is mutated, Asteroid1 might accumulate leading to another potentially harmful condition.

What is apparent from analyzing the genome data of human and mouse is that splice variants exist for the overlapped human *ATP2C1*, but not the mouse homolog. Recently, Morrissy *et al.* found an extensive correlation between antisense transcription and alternative splicing [16].

Although the reasons for the different pathologies caused by low levels of *ATP2C1* in human and mouse could be attributed to the differential species-specific cellular responses in dealing with alterations of the secretory pathway, these two possibilities are not mutually exclusive. A combination of species-specific gene regulation plus species-specific stress response mechanisms could underlie the different pathologies in human and mouse associated with SPCA1 deficiency.

## 3. *ASTE1* may Influence Expression of *ATP2C1* Isoforms

SPCA1 is a protein ubiquitously expressed in human tissues at different levels [17]. Different tissues may have different SPCA1 isoform pools, which will affect the speed, the efficiency and the type of protein secretion. The variability of protein expression levels is mostly relevant if we compare cancer *vs*. normal cells. Of interest, SPCA1 is highly expressed in human colon adenocarcinoma cells compared to normal tissue [18]. We also know that *ASTE1* has been found mutated in 80% of the colorectal cancers presenting frameshift mutations [15], and *ASTE1* frameshift mutations could play a key role in malignant transformation [15]. Furthermore, frameshift mutations in *ASTE1* generate a premature stop codon in the last exon [15]. Of consequence, reduced mature transcripts encoding for Asteroid1 can result in an increased activity in *ATP2C1* transcription, mainly the isoforms b–d. The inactivation of *ASTE1* and the high expression of SPCA1 in human cancer cells of the gastrointestinal tract are consistent with our model in which we propose that functional *ASTE1* has negative regulatory effects on *ATP2C1* expression, or at least on the isoforms b–d which encode regions overlapping with *ASTE1*.

**Figure 2:**
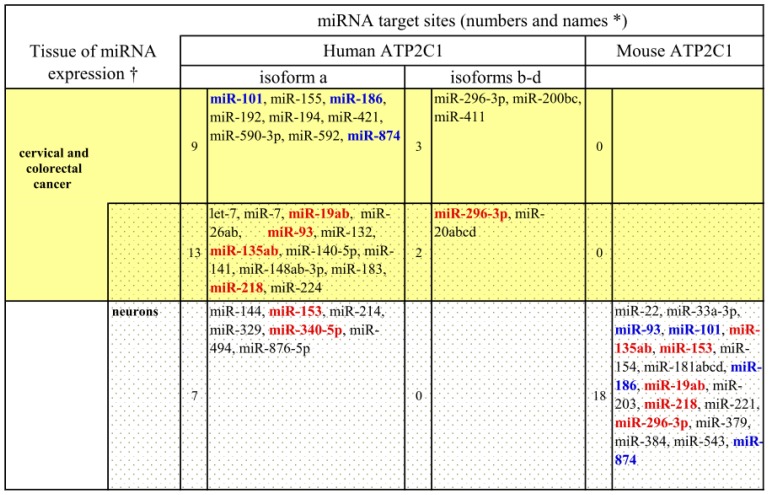
Predicted miRNA target sites in human and mouse in *ATP2C1* 3′UTRs and tissue-specific expression of the corresponding miRNAs. Red: conserved sites for miRNAs with conserved tissue expression. Blue: conserved sites, but different tissue expression of miRNA. The human *ATP2C1*a has a high number of target sites for cervical, colorectal and neural miRNAs compared to the isoforms b, c and d (29 *vs*. 5 sites). In the murine ortholog, the neural-specific miRNA regulation is conserved, but not the cervical/colorectal-specific one. miRNA predictions were obtained from TargetScan 6.0 (available online: www.targetscan.org (accessed on 5 December 2012)). Only the sites broadly conserved in all vertebrates and conserved in mammals were considered. The data for miRNA expression in tissues were obtained from smiRNAdb (available online: www.mirz.unibas.chcloningprofiles (accessed on 5 December 2012)),  microRNA.org and Landgraf *et al.* [20]. Only tissues discussed in the present article are shown in the figure. The cervical and colorectal cancer miRNAs are shown on a yellow background; the neuronal miRNAs, are shown on a gray background and the common miRNAs are shown on a merged yellow-gray background. († or dysregulation in pathological state; * Red: human-mouse conserved sites, same tissues of miRNA expression; Blue: human-mouse conserved sites, different tissues of miRNA expression).

Our speculation, which focuses on the *ATP2C1* alternative splicing and possibly its levels of expression affected by *ASTE1*, can be supported by a recent discovery where antisense transcription correlates with splicing and expression of sense genes in humans and all metazoans [16]. Namely, antisense transcripts may influence sense gene expression via a plethora of other mechanisms (*i.e.*, chromatin remodeling, transcriptional interference, RNA masking, double-stranded RNA-dependent mechanisms and translation interference) [19]. Interestingly, we noted that human *ATP2C1*a (which doesn’t overlap with *ASTE1*) has a high number of target sites for miRNAs which are differentially expressed in colorectal cancers, cervical cancers and normal colorectal cells. On the other hand, this is not the case for *ATP2C1* isoforms-b, c, and d (which do overlap with *ASTE1*) (Figure 2).

Combining these observations, we propose the following model: in normal tissues, *ASTE1* expression blocks the inclusion of the last SPCA1 exons (isoforms-b, c, d) and favors the formation of isoform-a, which in turn has a high number of target sites for colorectal specific miRNAs in its 3′UTR. This means that the level of SPCA1 in cervical and colorectal cells is tightly regulated via miRNA-mediated inhibition and any imbalance of miRNA expression (or of the composition of the pool of SPCA1 isoforms) will lead to pathological conditions. When *ASTE1* is down-regulated or mutated (e.g., in 80% of colorectal cancers), it no longer prevents the formation of the *ATP2C1* isoforms*-*b, c and d, which are less sensitive to miRNA-mediated inhibition. This is consistent with the observed high levels of SPCA1 in colon adenocarcinomas.

Consistent with the observation that Ca^2+^ ions along the secretory pathway play a pivotal role in neurons [21], *ATP2C1*a has a similar enrichment for target sites of neuronal-specific miRNAs compared to other human isoforms (20 *vs*. 2 target sites, respectively; Figure 2).

When analyzing the 3′UTR of the murine *ATP2C1,* we found substantial conservation of neural-specific miRNA target sites, but few colorectal and cervical-specific miRNA target sites. This might explain the different pathologies developed in human and mouse upon *ATP2C1* dysregulation.

Mutations in one allele of human *ATP2C1*, and thus low levels of the SPCA1 protein, cause a decrease in protein trafficking efficiency—enough for survival, but degenerating into HHD and rarely into cancer [12,13,22]. A speculative model has been recently proposed in which the presence of molecular or genetic modifiers either upstream or additional to *ATP2C1* mutation down-regulates expression of the functional allele of *ATP2C1* [23]. Loss of *ATP2C1* leads to an altered balance of ROS levels. As a consequence, there is an increased miR125b expression in keratinocytes, inducing differentiation and proliferation defects through suppression of targets, p63 and Notch1 [23], regulatory signaling components essential for the control of keratinocyte proliferation and differentiation. The compensatory presence of four *ATP2C1* isoforms in humans can explain how the remaining copies are sufficient to overcome, at least partially, the altered cell proliferation/differentiation and degeneration observed in HHD. On the contrary, loss of one copy in mouse increases the incidence of tumors [14], where only one isoform is not enough to overcome the deficiency and the intracellular Ca^2+^ signaling remains turned on, forcing the cell into a cancerous fate.

The different pathological outcomes in human and mouse could be explained by a difference in regulation of *ATP2C1*: in human, the transcription of *ASTE1* (partially overlapping with *ATP2C1*; Figure 1C) could influence the alternative splicing of *ATP2C1*, and of consequence the pool of *ATP2C1* isoforms, which display differential sensitivity to tissue-specific miRNAs. This, in turn, would lead to a tissue-specific regulation of SPCA1 protein levels. In mouse, where *ATP2C1* does not overlap with *ASTE1* (Figure 1D), nor is alternatively spliced, its regulation must be different, including its sensitivity to miRNAs. This could explain, at least in part, the different pathologies caused by *ATP2C1* mutations.

Based on these observations, we hypothesize a protective role of *ASTE1* on the regulation of the alternative splicing of the *ATP2C1* gene. Further evidence comes from 3D structure homology modeling by threading of the N-terminal side of Asteroid1 that reveals homology with Flap Endonuclease 1 (PDB access: 1UL1). If our hypothesis and modeling are correct, this implicates Asteroid1 in genomic stability, repair and replication with an indirect role in cell division, by regulating the physiological ratio of the different isoforms and inhibiting the (over-) expression of the *ATP2C1* gene. This in turn could prevent an abnormal subcellular localization of SPCA1, as is observed for the up-regulation of the related gene *ATP2C2* (encoding SPCA2, another member of this family of Ca^2+^-ATPase pumps) in breast cancer cells. In this case, SPCA2 appears to be associated with the plasma membrane, while it normally localizes along the secretory pathway [24]. However, SPCA1 over-expression may be a feature of the basal breast cancer subtype, which has the poorest prognosis [25]. The increased expression of SPCA1 (as well as SPCA2) may have a dramatic effect on the control of the cell cycle by activating cytosolic Ca^2+^ signaling and/or altering post-translational modification. Indeed, the Golgi apparatus (where SPCA1 localizes) functions as a trigger for entry into mitosis; it undergoes extensive fragmentation through a multistage process that allows its correct partitioning and inheritance by daughter cells. Strikingly, this Golgi fragmentation is required not only for inheritance but also for mitotic entrance itself [26]. Recent studies have demonstrated that severance of the ribbon into its constituent stacks during early G2 (at the precise stage of Golgi fragmentation) is the process controlling mitotic entry [26]. A dysregulation of Golgi fragmentation may lead to a block in mitotic entry, followed by apoptosis or necrosis. Alternatively, it could lead to aberrant cell division with possible oncogenic effects. Moreover, it has been recently reported that SPCA1 is strictly linked to the cytoskeleton in the regulation of membrane trafficking, and that alteration of the SPCA1 levels can affect the correct localization of SPCA1, altering protein secretion [27] and the maintenance of the Golgi apparatus [10,28], with reduced cell proliferation in SPCA1 depleted cells [10].

A further possible consequence of altered SPCA1 function is cytosolic Mn^2+^ intoxication. SPCA1 can also transport Mn^2+^ into the Golgi apparatus with the same affinity as Ca^2+^ [1,29]. A sufficient supply of Mn^2+^ is an absolute requirement for correct glycosylation of secretory proteins in the Golgi apparatus [30]. If SPCA1 is mutated and/or expression is altered the effect on the cell could be altered regulation of the apoptotic pathway [10].

Taken together, these observations support an additional, crucial role for SPCA1 in regulating Ca^2+^ concentration along the secretory pathway, which is critically involved in maintaining the Golgi apparatus as well as its major role of triggering TGN-to-plasma membrane trafficking through a possible interaction with the cytoskeleton, which thereby regulates the entry of cells into the G2 phase of mitosis. As a consequence of altered entry into mitosis, the cell can have an apoptotic or cancerous fate if SPCA1 is under- or over-functional, respectively.

## 4. Conclusions

Here, we hypothesize that human *ATP2C1* gene expression is influenced by an overlapping *ASTE1* gene. Experimental studies aiming to dissect the effects of this overlap on *SPCA1* transcription and/or alternative splicing in human cells will address this issue. Only in humans are SPCA1 isoforms seen and the role of each is poorly understood. Molecular, genetic, biochemical and ultra-structural studies could elucidate the levels and relative distributions of SPCA1 isoforms in normal as well as in HHD keratinocytes and cancers of the skin, breast and colon (and other SPCA1-related tumors). In detail, the generation of *ASTE1* knockout mice, if possible, could be useful to investigate possible effects on SPCA1 isoform pools in different tissues. Another approach consists of RNA interference of *ASTE1* in specific cell lines (*i.e.*, keratinocytes) and quantification of the expression of each SPCA1 isoforms by real-time PCR. To address our hypothesis of miRNA–mediated regulation of ATP2C1 expression, transient transfections in cultured cell lines could be performed to investigate the effects of over-expression or down-regulation of selected miRNAs on the expression of reporter genes containing ATP2C1 3′UTRs. Moreover, it would be interesting to analyze and compare the complete miRNA profiles of HHD-affected tissues and corresponding healthy tissues. The use of selective antibodies against each SPCA1 isoform would be invaluable to investigate their level of expression in these experimental conditions, both with light and electron microscopy and also protein quantification. From a clinical perspective, similar investigations can be conducted on biopsies from HHD patients. If the SPCA1 isoforms composition correlates with Asteroid1 expression, this will be in support of the proposed hypothesis. Additionally, *ASTE1* or its protein product Asteorid1 may provide an alternative/complementary diagnostic indicator for SPCA1-induced tumors in skin, breast and colon cancers—the main investigated SPCA1-related tumors.

## Abbreviations

Ca^2+^: calcium ions
HHD: Hailey-Hailey disease
SPCA1: secretory pathway (Ca^2+^)-ATPase pump type 1
ROS: reactive oxygen species
TGN: *trans*-Golgi network
